# Clinical significance of tumor-infiltrating lymphocytes in breast cancer

**DOI:** 10.1186/s40425-016-0165-6

**Published:** 2016-10-18

**Authors:** Sasha E. Stanton, Mary L. Disis

**Affiliations:** Tumor Vaccine Group, Center for Translational Medicine in Women’s Health, University of Washington, 850 Republican Street, 2nd Floor, Box 358050, Seattle, WA 98195-8050 USA

**Keywords:** Breast cancer, Tumor infiltrating lymphocytes, CD8 T-cell, FOXP3

## Abstract

Tumor infiltrating lymphocytes (TIL) play an essential role in mediating response to chemotherapy and improving clinical outcomes in all subtypes of breast cancer. Triple negative breast cancers (TN) are most likely to have tumors with >50 % lymphocytic infiltrate, termed lymphocyte predominant breast cancer, and derive the greatest survival benefit from each 10 % increase in TIL. The majority of HER2^+^ breast cancers have similar level of immune infiltrate as TN breast cancer yet the presence of TILs has not shown the same survival benefit. For HER2^+^ breast cancers, type 1 T-cells, either increased TBET^+^ tumor infiltration or increased type 1 HER2-specific CD4^+^ T-cells in the peripheral blood, are associated with better outcomes. Hormone receptor positive HER2 negative tumors tend to have the least immune infiltrate yet are the only breast cancer subtype to show worse prognosis with increased FOXP3 regulatory T-cell infiltrate. Notably, all breast cancer subtypes have tumors with low, intermediate, or high TIL infiltrate. Tumors with high TILs may also have increased PD-L1 expression which might be the reason that TN breast cancer seems to demonstrate the most robust clinical response to immune checkpoint inhibitor therapy but further investigation is needed. Tumors with intermediate or low levels of pre-treatment immune infiltrate, on the other hand, may benefit from an intervention that may increase TIL, particularly type 1 T-cells. Examples of these interventions include specific types of cytotoxic chemotherapy, radiation, or vaccine therapy. Therefore, the systematic evaluation of TIL and specific populations of TIL may be able to both guide prognosis and the appropriate sequencing of therapies in breast cancer.

## Background

Infiltration of immune cells, particularly infiltration of anti-tumor type 1 lymphocytes, has predicted improved prognosis in many different tumor types including colon, ovarian, lung and breast cancer [1–4]. Historically breast cancer was not thought to be immunologically active, particularly when compared to tumors such as melanoma. However recent evidence has emerged that tumor infiltrating lymphocytes (TILs) present in breast cancer prior to treatment can predict response to therapy and improved prognosis [4, 5].

Not only does the amount of lymphocytic infiltration but also the phenotype of that infiltrate determine clinical outcome. Type 1 T-cells are associated with favorable prognosis. CD4^+^ T-helper 1 (Th1) cells facilitate antigen presentation through cytokine secretion and activation of antigen presenting cells. CD8^+^ cytotoxic T-cells (CTL) are essential for tumor destruction [6]. On the other hand, type 2 CD4^+^ T-helper cells (Th2), including Forkhead box P3 (FOXP3) CD4^+^ regulatory T-cells, inhibit CTL function, support proliferation of B-lymphocytes, and may promote an anti-inflammatory immune response that could enhance tumor growth [7].

### Lymphocyte levels in breast cancer and prognosis

The adaptive immune response to breast cancer can be seen in infiltrating breast lesions as early as benign breast atypia and increases in density as invasive malignancy develops. In one retrospective study of 53 mastectomy samples, increased B-cell and T-cell immune infiltrate was identified in benign ductal hyperplasia, increased in ductal carcinoma *in situ* (DCIS), and was found in the greatest magnitude in invasive breast cancer [8]. In a study of 27 DCIS patients, all tumors demonstrated some level of TIL and 78 % of DCIS had >5 % infiltrate. High lymphocytic infiltrate was associated with young age and triple negative (TN) DCIS, similar to invasive cancer, with all TN DCIS (*p* = 0.0008) having programed death-ligand 1 (PD-L1) expression [9]. The phenotype of the T-cell response has also been shown to predict prognosis in DCIS. In a study of 62 DCIS samples, FOXP3^+^ infiltrate above the mean predicted decreased relapse free survival (RFS) (HR 2.8; 95 % CI 0.99–7.99, *p* = 0.05) [10]. Conversely, increased expression of a Th1 gene signature predicted improved survival in 31 patients with DCIS [11]. Tumor lymphocytic infiltrate may be able to be developed for use to stratify risk of recurrence and need for aggressive therapies in DCIS, and immune therapies may provide well-tolerated approaches to explore for improved DCIS treatment [12].

In invasive breast cancer, the greatest clinical benefit is seen in tumors with >50 % lymphocytic infiltrate (lymphocyte predominant breast cancer (LPBC)). In patients with locally advanced breast cancer treated with neoadjuvant chemotherapy, patients with LPBC had a 40 % pathologic complete response (pCR) (OR 1.38, *p* = 0.012 95 % CI 1.08–1.78) as compared to 7 % pCR in patients with tumors that had no lymphocytic infiltrate [4]. Increased CD8^+^ T-cells have also been shown to predict improved clinical outcome, with higher intratumoral CD8^+^ T-cell infiltrate associated with improved breast cancer specific survival (HR 0.55 95 % CI, 0.39 to 0.78 *p* = 0.001) in one large study of 1334 patients [13]. This has not been replicated in other clinical studies [14–16]. Infiltration of TBET^+^ cells (T-box transcription factor TBX21, a marker of type 1 T-cells) can also predict improved disease free survival (DFS) in all breast cancer subtypes with breast cancer patients with tumors containing < 30 TBET^+^ cells having decreased DFS as compared to patients with tumors containing ≥30 TBET^+^ cells (RR 5.62 95 % CI 1.48–50.19 *p* = 0.0027 *n* = 617) [17]. On the other hand, the presence of the Th2 marker FOXP3^+^ in the tumor has been associated with worse prognosis. In an evaluation of over 200 breast cancers, patients with tumors containing greater than 15 FOXP3^+^ cells had decreased RFS (*p* = 0.04 HR 1.58, 95 % CI 1.01 to 2.47) and overall survival (OS) (*p* = 0.07, HR 1.62 95 % CI 0.96 to 2.74) [10]. Even when examining all breast cancer subtypes together, the composition and magnitude of the tumor immune infiltrate affects clinical outcome and demonstrates that breast cancer is an immunogenic tumor. However the impact of TILs on clinical outcome is most evident when the breast cancer subtypes are evaluated separately.

**Fig. 1:**
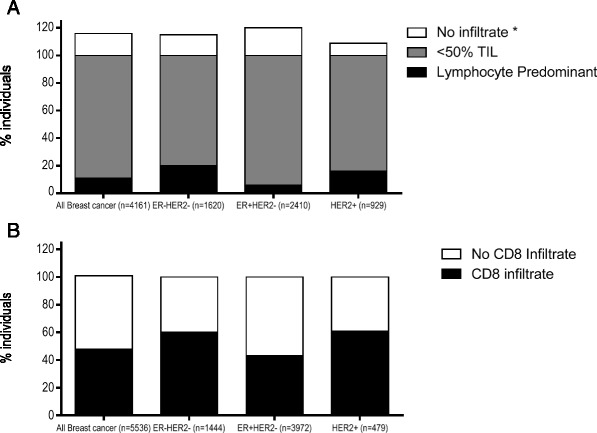
Most breast cancers have evidence of lymphocytic infiltrates at the time of diagnosis, although the level of infiltrate is modest, and the presence of CD8^+^ infiltrate varies between the breast cancer subtypes. The % individuals (x-axis) are shown for: **a** no evidence of TIL (*white*), TIL <50 % (*medium grey*), and LBPC (*black*) data compiled from 6 studies. *Only one to two studies evaluated no infiltrate separately. **b** Presence of CD8^+^ infiltrate (*black*) or no CD8^+^ infiltrate (*white*), data compiled from 3 studies

In HER2^+^ and TN breast cancer, even incremental increases in TILs both in and surrounding the tumor have shown to predict both response to chemotherapy and improved survival in patients [5, 18–20]. Furthermore, LPBC is more common in both TN and HER2^+^ breast cancers, with a median of 20 % TN tumors and 16 % HER2^+^ tumors having LPBC (Fig. 1a) [21]. One study of 256 TN tumors demonstrated every 10 % increase in TIL correlated with a 17 % decrease in the risk of recurrence (*p* = 0.023, HR 0.83; 95 % CI 0.71–0.98) and a 27 % decreased risk of death (*p* = 0.035, HR 0.73; 95 % CI 0.54–0.98) [5]. Similarly, for each 10 % increase in stromal TIL there was 18 % increase in OS (HR 0.82 95 % CI 0.69–0.96) in 112 HER2^+^ breast cancer patients [20]. For both HER2^+^ and TN breast cancer, while the best response has been seen in LPBC that have the highest infiltrate, even small increases in TIL lead to incremental increases in improved survival and may suggest that even therapies that modestly increase TIL can benefit clinical outcome in these subtypes.

Both TN and HER2^+^ patients have evidence of CD8^+^ T-cell infiltrate with approximately 60 % of tumors containing CD8^+^ T-cells (Fig. 1b) [21]. CD8^+^ infiltrate has only been shown to predict a survival benefit in TN breast cancer; improved breast cancer specific survival was seen with any intratumoral CD8^+^ infiltrate (*p* = 0.001, HR 0.35; 95 % CI 0.23 to 0.54 *n* = 927) (Table 1) [15]. While intratumoral CD8^+^ T-cells do not predict improved clinical outcome in HER2^+^ breast cancer, TBET^+^ tumor infiltrate predicted improved RFS (*p* = 0.04 HR 4.76, 95 % CI 1.07 to 20) in 102 HER2 tumors treated with trastuzumab [22]. For HER2^+^ breast cancer, the effect of CD8^+^ tumor infiltrate may require hormone positive HER2^+^ tumors to be evaluated separately from hormone negative HER2^+^ tumors. The only study that stratified HER2^+^ tumors by hormone receptor status found that CD8^+^ tumor infiltrate was associated with RFS (*p* = 0.041) (*p* = 0.064, HR 0.75 % CI 0.51–1.11 *n* = 227) in hormone receptor negative HER2^+^ breast cancer but not hormone receptor positive HER2^+^ breast cancer [15]. These data suggest that the immune infiltrate in HER2^+^ breast cancer may be more influenced by hormone receptor status rather than HER2 protein overexpression.

**Table 1:** Effect on outcome of LPBC, CD8+, or FOXP3 tumor infiltrate by subtype

	Breast cancer subtype					
	TN		HR+		HER2+	
Immune infiltrate	DFS or RFS	OS or DSS	DFS or RFS	OS or DSS	DFS or RFS	OS or DSS
LPBC	+++	+++			++	+
Elevated CD8+	+	+++		+	+ (TBET)	
Elevated FOXP3			-	-		

*Abbreviations*: *DFS* disease free survival, *RFS* relapse free survival, *OS* overall survival, *DSS* disease specific survival, *LPBC* lymphocyte predominant breast cancer, *TN* triple negative, *HR* hormone receptor

+++ Increased (>2 sources); ++ (increased 2 sources) + Increased (one source); - Decreased (one source)

As compared to TN or HER2^+^ subtypes, hormone receptor positive HER2 negative (HR) tumors both have less TIL and the tumors with LPBC do not show the same improved survival benefit. Only 6 % of HR tumors have LPBC and less than half have CD8^+^ T-cell infiltrate (Fig. 1) [21]. The decreased lymphocytic infiltrate may be due to the expression of the estrogen receptor which has been shown to both promote a Th2 immune environment and decrease MHC class II expression in breast cancer cells [23, 24]. However, HR breast cancer is the only breast cancer subtype where FOXP3^+^ infiltrate predicts a worse survival [10, 21, 25]. In 148 HR^+^ tumors, increased FOXP3^+^ infiltrate was associated with a decreased RFS (*p* = 0.006 HR 2.20 95 % CI 1.26–3.85) and OS (*p* = 0.006, HR 2.57 95 % CI 1.31–5.60) [10]. Potentially, a therapy that can effectively decrease FOXP3^+^ infiltrate may increase the magnitude of lymphocytic infiltrate in HR tumors and may improve clinical response in the neoadjuvant setting (Table 2).

**Table 2:** Biomarker staining by IHC and prognosis in breast cancer subtypes

Marker	Measurement	Good prognosis	Breast cancer subtype
CD8	Presence/absence	Presence	TN
PD-L1	>5 % membrane staining	Presence	TN
HER2	1+, 2+, 3+	1+	HER2^+^
FOXP3	Low/High (above and below median)	Low	HR^+^

*Abbreviations*: *TN* triple negative, *HR* hormone receptor

### Immune checkpoint inhibitor therapy in breast cancer

PD-L1 expression has been associated with increased TILs and better prognosis in breast cancer. In a study of 45 primary breast cancers, 89 % PD-L1^+^ and 24 % PD-L1^-^ breast cancers had moderate or diffuse TILs. Furthermore, none of patients that had PD-L1^+^ breast cancer at diagnosis developed distant recurrence whereas 15 % of the patients that had PD-L1^-^ breast cancer at diagnosis did develop distance recurrence [26]. PD-L1 infiltrate has been associated with TN breast cancer and CD8+ T-cell infiltrate (Table 2) [27]. These data suggest that PD-L1 expression is a marker of an immunologically active breast cancer. Although increased TIL has also been associated with increased PD-L1 infiltrate, the association between increased TIL and response to immune checkpoint therapy has not yet been established [28, 29]. Early trials of immune checkpoint inhibitor specific monoclonal antibodies have shown only modest clinical efficacy in breast cancer. None of the breast cancer patients included in the initial pembrolizumab (anti-PD-1) trial showed any response to treatment and the combination of tremelimumab (anti-CTLA4) and exemestane in HR metastatic breast cancer demonstrated development of stable disease as best response in 42 % of patients [30, 31]. Several studies have shown a modest clinical response in TN breast cancer to pembrolizumab and atezolizumab (anti-PD-L1) inhibitor monotherapy, including some complete responders. The Keynote 012 trial reporting 27 patients with PD-L1 positive metastatic TN breast cancer treated with pembrolizumab as a monotherapy showed an overall response rate of 19 % with one complete response and four partial responses as well as 26 % patients with stable disease [32]. Similar results have been seen using anti-PD-L1 monoclonal antibodies. A trial of 21 metastatic TN breast cancer patients treated with atezolizumab monotherapy demonstrated a 19 % overall response rate with two complete responses and two partial responses [33]. Early data has further demonstrated that combining chemotherapy and checkpoint inhibitor therapy may increase the number of clinical responses to immune checkpoint inhibitor therapy in TN breast cancer. In a study of 24 metastatic TN breast cancer patients, the combination of avelumab (anti-PD-L1) inhibitor and nab-paclitaxel showed a response rate of 42 % (95 % CI 22.1 to 63.4 %) including a complete response rate of 4 %, partial response rate of 67 %, and stable disease in 21 % of patients [34]. This data is promising, despite only 12 months of follow up, that use of checkpoint inhibitors in combination with chemotherapies may expand the number of breast cancer patients that respond to immune checkpoint inhibitor therapies particularly in TN breast cancer.

The number of patients with HER2^+^ and HR breast cancer subtypes who respond to immune checkpoint inhibitor therapy is much lower. In one study of 27 HER2^+^ patients and 72 HR patients receiving avelumab therapy, only 4 % of HER2^+^ and 3 % of HR patients demonstrated a clinical response [35]. In one study of 25 PD-L1 positive HR breast cancer patients treated with pembrolizumab, an overall response rate of 12 % was observed and these were only partial responses [36]. Newer immune checkpoint therapies that activate the T-cell immune response rather than block inhibition of T-cell activity including OX40 (CD134), OX40 ligand, and 41BB (CD137) may be able to enhance immune-associated anti-tumor activity in breast cancer. In pre-clinical mouse mammary tumor models, treatment with either OX40 or 41BB monoclonal antibodies were able to significantly decrease both tumor growth and development of metastases [37–39]. Several clinical trials using combination checkpoint therapy are currently ongoing.

### Augmenting immunity through conventional breast cancer chemotherapy and monoclonal antibody therapy

A major mechanism of action of trastuzumab therapy in HER2^+^ breast cancer may be immunologic. Monoclonal antibodies can trigger antibody dependent cell mediated cytotoxicity (ADCC) that results in the activation of NK T-cells, macrophages, and dendritic cells. Activation of cells of the innate immune system leads to the secretion of Th1 cytokines, enhanced antigen processing, and presentation of endogenous tumor antigens to T-cells eliciting an adaptive immune response [40, 41]. Furthermore, the enhanced HER2 specific immunity associated with trastuzumab therapy has been associated with improve clinical prognosis. In a study of 87 locally advanced HER2 breast cancer patients treated with trastuzumab, 94 % of the patients with high HER2 specific interferon gamma (IFN-g) Th1 immunity had pCR as compared to 33 % of patients that did not achieve pCR (*p* = 0.0002). In multivariate analysis, a high HER2-specific Th1 immune response predicted whether a patient would develop pCR (OR 8.82 95 % CI 1.50 to 51.83 *p* = 0.016) [42]. In an adjuvant chemotherapy trial of 95 HER2 breast cancer patients, high HER2-specific Th1 immunity predicted improved RFS (HR 16.9 95 % CI 3.9 to 71.4 *p* < 0.001) [43]. Both of these studies found that trastuzumab was needed to stimulate increased Th1 HER2 specific immune responses as patients not treated with trastuzumab did develop the high Th1 HER2 specific immunity. Similarly in the FINHER study of 209 HER2 breast cancer patients, only patients that had been treated with trastuzumab had improved distant DFS with each 10 % increase in TIL (HR 0.82 95 % CI 0.58 to 1.16, *p* = 0.025 *n* = 94) [19]. For HER2^+^ breast cancer, the immunologic function of trastuzumab to induce type 1 immunity appears to be important for its therapeutic efficacy.

Cytotoxic chemotherapy has also been shown to increase type 1 T-cell response. Some chemotherapeutic agents have been shown to trigger immune recognition of the tumor by induction of stress proteins released during cell death. For example doxorubicin induces the secretion of a protein called high-mobility-group box 1 (HMGB1) from dying cancer cells that binds to toll-like receptor (TLR) 4 on dendritic cells resulting in the secretion of IFN-g, antigen presentation, and activation of T-cells [44]. Toll-like receptors are highly conserved pattern recognition receptors that activate the immune recognition and enhance pathogen presentation to the adaptive immune system [45]. This resulting adaptive immune response may be a major mechanism of response to doxorubicin therapy because a TLR-4 genetic polymorphism, Asp299Gly, has been shown to decrease the binding of HMGB1 and IFN-g secretion by 50 % (*p* < 0.05) in in vitro assays. In an evaluation of 280 breast cancer patients treated with adjuvant doxorubicin, 40 % of the patients carrying the TLR-4 Asp299Gly polymorphism developed metastatic disease in 5 years as compared to 27 % of patients without the polymorphism (RR 1.53 95 % CI 1.1 to 3.59 *p* = 0.03) [44]. When comparing gene expression in 114 breast cancer patients that received anthracycline chemotherapy and 1062 breast cancer patients that did not receive chemotherapy, anthracycline therapy increased type 1 immune response, and the increased CD8^+^ (HR 0.72 95 % CI 0.59 to 0.82 *p* = 0.005) and IFN-g (HR 0.56 95 % CI 0.56 to 0.89 *p* = 0.016) expression was associated with improved pCR in patients that had been treated with anthracycline [46]. Paclitaxel has also been shown to increase tumor infiltrating type 1 T-cells by increasing the expression of type 1 cytokines and decreasing Th2 CD4^+^ T-cells in the tumor [47, 48]. Cyclophosphamide has been shown to decrease Th2 regulatory T-cells without decreasing circulating Th1 immune response at low doses [49]. Carboplatin and cisplatin have been shown to increase MHC class 1 expression on the tumor while also decreasing intratumoral myeloid derived suppressor cells and Th2 regulatory T-cells in the tumor [50]. Studies are ongoing to determine the most effective way to dose or sequence these agents to optimize their immunologic effects.

### Newer options for immune modulation in breast cancer therapy

Early clinical trials of metastatic breast cancer have demonstrated that localized therapies, including radiation, cryoablation, and cellular stress signals such as TLR agonists, both induce local destruction of the tumor as well as increase the systemic anti-tumor immune response demonstrating clinical response in tumors distant from the treated lesion. These distant responses occur because the local cellular damage increases cellular stress signals and trigger type 1 cytokine release, recruiting antigen presenting cells to the tumor and improving antigen presentation of tumor antigens to T-cells converting the tumor an *in situ* vaccine [51, 52]. In a trial of 41 metastatic solid tumor patients treated with radiation and concurrent adjuvant granulocyte-macrophage colony-stimulating factor, 11 of 41 patients (26.8 %, 95 % CI 14.2 to 49.9) had a 30 % reduction in the volume of non-irradiated tumors. Five of the 11 responding patients had breast cancer [53]. Similarly, cryoablation of breast tumors has been shown to increase type 1 cytokine secretion resulting in enhanced presentation of tumor-specific antigens to T-cells inducing a tumor-specific T-cell response [54, 55]. Cryoablation is currently in clinical trials along with ipilimumab in breast cancer and has shown both increase effector T-cell to regulatory T-cell ratio and increase T-cell clonal expansion in the tumor [56]. The TLR7 agonist imiquimod has been shown to induce partial response in 20 % (95 % CI 3 to 56 %) of 10 breast cancer patients with skin metastases that are typically unresponsive to therapy [57]. For tumors with low immune infiltrate, local therapies can increase the systemic T-cell response against the tumor and therefore increase the anti-tumor immune response to areas of disease distant from the therapy.

## Conclusion

With evidence that the magnitude and composition of tumor immune infiltrate can affect prognosis and response to therapy both for DCIS and invasive cancer, the pre-therapy tumor immune environment can be used both as a biomarker for the prognosis of an individuals’ disease as well as a guide to determine what is the most appropriate therapy. Currently, the International TILs Working Group has started standardizing evaluation of breast cancer TILs to be able to use this in clinical practice [58]. Standardizing how to characterize a breast tumor by both the subtype and immune environment (having high, intermediate, or low immune infiltrate) will allow both the identification of patients that may only need treatment with various emerging immune therapies (including checkpoint inhibitor therapy) and provide the optimal combinations and timing of these powerful therapies to the patients with lower immune infiltrate to allow a wider population of breast cancer patients to benefit from targeted immune therapy.

## Abbreviations

DCIS: Ductal carcinoma *in situ*
DFS: Disease free survival
HER^+^: HER2 positive
HR: Hormone receptor positive HER2 negative
IFN-g: Interferon gamma
LPBC: Lymphocyte predominant breast cancer
OS: Overall survival
pCR: Pathologic complete response
RFS: Relapse free survival
Th1: Type 1 helper T-cells
Th2: Type 2 helper T-cells
TIL: Tumor infiltrating lymphocytes
TN: Triple negative
